# Mastering your fellowship: Part 2, 2024

**DOI:** 10.4102/safp.v66i1.5866

**Published:** 2024-02-14

**Authors:** Mergan Naidoo, Klaus von Pressentin, Andrew Ross, Selvandran Rangiah

**Affiliations:** 1 Department of Family Medicine, College of Health Sciences, University of KwaZulu-Natal, Durban, South Africa; 2 Department of Family Community and Emergency Care, Faculty of Health Sciences, University of Cape Town, Cape Town, South Africa; 3 Department of Family Medicine, Faculty of Medicine, University of KwaZulu-Natal, Durban, South Africa; 4 Department of Family Medicine, Faculty of Health Sciences, University of KwaZulu-Natal, Durban, South Africa

**Keywords:** family physicians, Fellowship of the College of Family Physicians of South Africa examination, family medicine registrars, postgraduate training, national exit examination, emergency care

## Abstract

The ‘Mastering your Fellowship’ series provides examples of the question format encountered in the written and clinical examinations, Part A of the Fellowship of the College of Family Physicians of South Africa (FCFP [SA]) examination. The series is aimed at helping family medicine registrars prepare for this examination.

This section in the South African Family Practice journal is aimed at helping registrars prepare for Part A of the Fellowship of the College of Family Physicians of South Africa [FCFP (SA)] examination and will provide examples of the question formats encountered in the written examination: multiple choice question (MCQ) in the form of single best answer (SBA – Type A) and/or extended matching question (EMQ – Type R); short answer questions (SAQ), questions based on the critical reading of a journal (evidence-based medicine) and an example of an objectively structured clinical examination (OSCE) question. Each of these question types is presented based on the College of Family Physicians blueprint and the key learning outcomes of the FCFP programme. The MCQs are based on the 10 clinical domains of family medicine, and the SAQs will be aligned with the five national unit standards. The critical reading section will include evidence-based medicine and primary care research methods.

This edition is based on unit standard one (critically reviewing new evidence and applying the evidence in practice, leadership and governance), unit standard two (evaluate and manage a patient according to the biopsychosocial approach) and unit standard five (conduct all aspects of healthcare in an ethical, legal and professional manner). The domain covered in this edition is Anaesthetics. We suggest you attempt to answer the questions (by yourself or with peers or supervisors) before finding the model answers online: https://www.safpj.co.za/.

Please visit the Colleges of Medicine website for guidelines on the Fellowship examination: https://www.cmsa.co.za/view_exam.aspx?QualificationID=9.

We are keen to hear about how this series assists registrars and their supervisors in preparing for the FCFP (SA) examination. Please email us (naidoom@ukzn.ac.za) your feedback and suggestions.

## Multiple choice question (MCQ): Single best answer

A 30-year-old female needs an emergency caesarean delivery (CD). She had no comorbidities; her baseline blood pressure (BP) was 124/72 mmHg, her pulse rate was 74 beats per min (bpm) and she had an American Society of Anaesthesiologists (ASA) rating of one. She has a 39-week gestation, and her CD is being performed for foetal distress. A few minutes after the spinal anaesthetic, she developed a pulse rate of 38 bpm. Her BP = 120/84 mmHg, and she reports no paresthesias in her upper limbs. Her sensory level for the block is assessed as thoracic level 6. What is the most appropriate next step?

- Administer adrenaline
- Administer atropine
- Administer ephedrine
- Administer phenylephrine
- Intubate and ventilate the patient

Short answer: b)

Severe bradycardia is a pulse rate of less than 40 beats per min (bpm) and is associated with an increased risk of asystole during neuraxial anaesthesia in healthy individuals. Approximately 10% of patients develop bradycardia below 50 bpm during neuraxial anaesthesia. The incidence of bradycardia is significantly associated with low baseline heart rate (BHR), ASA physical status 1, preoperative use of adrenergic blocking agents (beta-blockers) and high-peak sensory levels. Surprisingly, younger patients with low anaesthetic risks were affected equally by this phenomenon compared with older individuals.

The paradoxical effect of increasing cardiac inhibitory neural activity from an underfilled hypercontractile left ventricle is frequently referred to as the Bezold–Jarisch reflex (BJR). Neuraxial anaesthesia can increase cardiac vagal tone and decrease venous return. Reduced venous return stimulates cardiac receptors with increased parasympathetic activity and inhibition of sympathetic activity, manifesting as a classical BJR triad of hypotension, bradycardia and peripheral vasodilation. The decreased venous return to the heart during neuraxial anaesthesia initiates several reflexes that precipitate bradycardia through a spared parasympathetic nervous system. The blockade of cardiac accelerator fibres often occurs when the level of sympathetic blockade extends above T4. However, some researchers reported heart rates of less than 30 bpm among patients who had peak sensory block levels between T4 and T8. This may be explained by the fact that the level of sympathetic blockade may extend as much as six levels higher. Ventricular mechanoreceptors located primarily in the inferior-posterior ventricular wall (nonmyelinated C-fibres) may initiate the bradycardia reflex. The actual cause of the bradycardia is not well understood.

In the above-mentioned case scenario, the patient developed severe bradycardia in response to the spinal anaesthesia but maintained her blood pressure. This may just be an early phase of the BJR so appropriate interventions at this stage would be to administer atropine and continue monitoring the vital signs. If the patient does not respond, ephedrine and adrenaline may need to be administered together with intravenous fluids. Preloading patients undergoing spinal anaesthesia with fluids before anaesthesia and being aware that patients with low basal heart rates may develop severe bradycardia provides an awareness of possible complications. Bradycardia can develop at any time during the anaesthesia including during the recovery period so vigilant monitoring of vital signs is of paramount importance.

### Further reading

- Lesser JB, Sanborn KV, Valskys R, Kuroda M. Severe bradycardia during spinal and epidural anesthesia recorded by an anesthesia information management system. Anesthesiology. 2003;99(4):859–866. https://doi.org/10.1097/00000542-200310000-00018
- Torluttler M, Van Hoving N, Lomas V. How to administer a spinal anaesthetic. In: Mash B, Brits B, Naidoo M, Ras T, editors. South African family practice manual. 4th ed. Pretoria: Van Schaik Publishers; 2023; p. 530–534.

## Short answer question (SAQ): The family physician’s role as a leader of clinical governance in the domain of emergencies and trauma

You are the family physician working in a rural district hospital. The hospital has 250 beds and is staffed with 12 medical officers (8 posts are vacant because of a lack of funding). Around 300 deliveries are performed a month in the hospital. Last month there was a maternal death because of a high spinal and failed intubation. The district and provincial assessors declared the death as avoidable.

Details of the case were: Caesarean delivery (CD) for foetal distress at 2:00 am. Patient was Gravida 3 Parity 2, with six antenatal visits. Clinically she appeared obese with a short neck. Following delivery of the spinal anaesthetic the patient developed respiratory arrest. Neither the Community Service Medical officer (CSMO) responsible for the anaesthetic nor the medical officer (MO) who was doing the caesarean delivery was able to intubate or ventilate the patient and she died from hypoxic brain injury.

### 1. List and justify 4 issues that could have contributed to the poor outcome in this scenario. (Any 4 points)

- Inadequate skills mix in theatre with a lack of training or competency measurement of staff (one of the doctors on call should be able to give a general anaesthesia for a high spinal).
- Inadequate pre-anaesthetic risk assessment of the patient (anticipation and preparation for difficult intubation – risk factors such as obesity and short neck need to be observed, in addition to the usual pregnancy risks for difficult intubation).
- Inadequate preparation in theatre in terms of drugs and equipment in case of difficult intubation. A difficult intubation trolley should be available with different size endotracheal tubes, introducers and a gum elastic bougie (GEM) for difficult intubation. A laryngeal mask airway (LMA) and reversal drugs available should also be available following induction of anaesthesia (need to carefully consider mode of anaesthesia before delivering the anaesthetic in high risk cases and not give long-acting muscle relaxants until the patient is safely intubated), bag-mask ventilation (BMV) patient until help arrives.
- Inadequate process for calling for backup in an emergency (ideally should have back up for both CD and anaesthetic 24/7 with someone who can be in theatre within 10 min if the need arises).
- Shortage of staff (8 posts vacant), which increases pressure on staff and burn-out.

### 2. What can you do to improve the individual capability of the doctor(s) involved?

(Any 4 points – clinical governance activities that address individual capability of doctors should be applied in this question. May include examples of capacity building/training/mentoring and risk management/assess competence of new clinical staff/morbidity and mortality (M&M’s) and application of clinical guidelines)

- All staff need to be assessed for competency before being left to work independently. This can be performed by working alongside a senior doctor in theatre receiving ‘bedside’ training and assessment, keeping a skills logbook.
- Small group teaching/skills lab could be used to ensure training on pre-anaesthetic (airway assessment and management), anaesthetic (mode of anaesthesia and anaesthetic plan, as well as how to convert to GA from spinal anaesthesia) and post-anaesthetic management.
- Bedside/small group teaching/skills lab should be used to train all staff for emergency scenarios – e.g. convert to GA for failed spinal, approach to the difficult airway, management of haemodynamic changes and the use of vasopressors during spinal anaesthetic for CD.
- Rigorous training in theatre preparation – ‘always be prepared’ – e.g. anaesthetic machine check, appropriate equipment readily available and functioning, emergency drugs available and drawn up and ready for use. This includes easy access to a difficult intubation trolley with key equipment (ET tubes with introducer, gum elastic bougie, Macintosh laryngoscope, LMA, etc.).
- Provide and implementation of clinical guidelines.
- Carry out regular M&M’s so that things can be prevented from happening again.
- Arrange for additional anaesthetic training in the regional hospital or arrange regular visits from anaesthetist on the district clinical specialist teams (DCST).
- Encourage staff to call for help (even in the middle of the night). Provide mentoring and support to ensure that staff know that they can call for help when they suspect that a patient might have a difficult airway.

### 3. What can you do to improve the way things are organised in the facility to prevent such an event recurring?

(Any 4 points – clinical governance activities that address organisation in the facility should be applied in this question. May include activities that help to solve problems in teams including root cause analysis, fishbones, audits and quality improvement cycles, improving patient safety and M&M’s, revision of guidelines and critically reviewing new evidence)

- Skills audit of MOs and accredit MOs with anaesthetic skills to the roster to ensure correct skills mix on the on-call roster
- Ensure that a senior with anaesthetic experience is available 24/7 to provide additional support to those in theatre
- Regular audits of emergency equipment and the difficult airway trolley with key equipment being available – ET tube with introducer, gum elastic bougie, Macintosh laryngoscope in theatre
- Run regular drills on the management of the difficult airway
- Ensure regular continuing medical education (CME)/training on airway management
- Facilitate regular review of difficult anaesthetics with key learning outcomes with all staff – M&M’s

### 4. The CSMO is distraught at the outcome and is considering resigning and leaving medicine. As a family physician how would you help the CSMO to deal with this medical mistake? (Any 8 points)

- Arrange a debriefing with the CSMO to understand what happened and reasons for the decisions that were made.
- Allow for the ventilation of thoughts, emotions and experiences associated with the event.
- Encourage CSMO to admit/acknowledge mistakes that were made and face the reality of what happened.
- Help him/her identify where he/she went wrong so as to prevent future error (use of the Gibbs reflective cycle might be a useful tool to encourage reflection and leaning from this experience).
- Explore motives – was the CSMO trying to do the right thing at the time?
- Arrange professional counselling if necessary.
- Encourage him/her to talk to trusted peers and non-medical friends.
- Explore why he/she did medicine and explore his/her personal values, vision and purpose.
- As the incident is serious encourage the CSMO to immediately draft a statement that factually sets out what happened and his/her involvement.
- Discuss the need for disclosure to the family.
- Any other appropriate responses.

### Further reading

- Anaesthesia for Caesarian Delivery. Portfolio of learning. Fellowship of the College of Family Physicians of South Africa. [cited 2023 July 1]. Available from: https://www.cmsa.co.za/view_exam.aspx?QualificationID=9
- Viljoen V. How to deal with medical mistakes. In: South African family practice manual. 4th ed. Pretoria: Van Schaik Publishers; 2023; p. 776–780.

## Critical appraisal of research

Read the accompanying article carefully and then answer the following questions. As far as possible, use your own words. Do not copy out chunks from the article. Be guided by the allocation of marks concerning the length of your responses.

- Ernstzen D, Keet J, Louw KA, et al. ‘So, you must understand that that group changed everything’: Perspectives on a telehealth group intervention for individuals with chronic pain. BMC Musculoskelet Disord. 2022;23(1):1–6. https://doi.org/10.1186/s12891-022-05467-7

**Total: 30 marks**

1. Did the study address a clearly focused question? Discuss. (3 marks)
2. Explain why a qualitative research methodology may be most appropriate for this research question. Comment on the authors’ choice of qualitative study design. (5 marks)
3. Critically appraise the authors’ choice of participant sampling criteria. (5 marks)
4. Critically appraise the participant recruitment strategy. (5 marks)
5. Were the participants included in the study well described? Justify your response. (4 marks)
6. How did the researchers ensure trustworthiness with regard to their approach to qualitative data analysis? Use the following concepts to structure your answer: credibility, transferability, dependability, audit trails and reflexivity. (8 marks)

**Suggested answers:**

1. **Did the study address a clearly focused question? Discuss. (3 marks)**
  - The authors aimed to explore the acceptability and feasibility of a telehealth group intervention in the South African context to understand the knowledge gap of such an intervention in the local context. The experiences of the patients are presented in this article and a separate article reports on the experiences of the providers.
  - One may assert that the question is reasonably focused as it describes the population of interest (the patients who participated in the telehealth group intervention) and the condition or phenomenon of interest (the efficacy of the telehealth group intervention) in a particular community or area (the South African context).
  - The contextual description is rather broad as the participants were drawn from the drainage area of two tertiary metropolitan hospitals based in Cape Town, Western Cape. However, this broad study frame may be deemed acceptable given the specialised nature of the service providing the intervention.
2. **Explain why a qualitative research methodology may be most appropriate for this research question. Comment on the authors’ choice of qualitative study design. (5 marks)**
  - Qualitative research aims to understand meaning, such as the meanings that people attribute to their work, their behaviours or beliefs or their attitudes or perceptions.
  - For this study, the authors employed a qualitative, exploratory descriptive design with a phenomenological approach. The authors wished to explore the phenomenon of interest from the perspective of the participants who engaged with and completed a telehealth group programme.
  - This supports the choice of using a qualitative approach compared with a quantitative approach, which aims to develop objective theories by generating quantifiable numerical data.
  - The authors described their qualitative study design as exploratory using individual interviews structured with an interview guide. Exploratory studies aim to generate new knowledge by exploring novel topics where little or no data exist. These exploratory study designs include quantitative (surveys) as well as qualitative research methods (participant observation, interviews and focus groups).
  - In this article, the authors stated their philosophical perspective and its alignment with their choice of research methodology: they chose a phenomenological approach to gain insight into the participants’ lived experience of and their perspectives regarding participating in a telehealth group programme for chronic pain. Therefore, the authors adhered to the consolidated criteria for reporting qualitative studies (COREQ) reporting guideline that states that the methodological orientation should be stated to underpin the study.
3. **Critically ap praise the authors’ choice of participant sampling criteria. (5 marks)**
  - The researchers employed a type of purposive sampling method called criterion sampling, as they used inclusion and exclusion criteria to choose the participants who will potentially be the most informative in terms of their ability to answer the study question.
  - Table 1 (Ernstzen et al. 2022) in the article contains the inclusion and exclusion criteria. The first inclusion criteria one combines three inclusion criteria, namely being an adult, having a confirmed diagnosis of non-cancerous pain and having been referred by the treating physician. This combined criterion is aligned with the referral criteria for the existing Pain Education Empowerment Programme (PEEP) intervention. Pending investigations was a linked exclusion criterion, as a confirmed and final diagnosis was needed prior to referral for the intervention.
  - The second inclusion criterion, evaluated as being safe to engage in moderate to vigorous physical activity was informed by the American College of Sports Medicine guidelines. This is reasonable, as the telehealth programme involved physical exercises in the home environment without the onsite presence of a therapist to intervene if needed.
  - The third inclusion criterion is linked to the second exclusion criterion, namely both access to and confidence in using an electronic platform via a smartphone. The authors added a footnote to Table 1 in the article (Ernstzen et al. 2022), which acknowledges that these criteria may have excluded potential participants who did not have access to a smartphone.
  - This selection bias is reasonable, however, given the electronic nature of the telehealth intervention and the fact that the authors wished to evaluate the feasibility and acceptability of the intervention. The authors acknowledged the need for future research, which will explore issues around smartphone access and digital literacy when implementing telehealth interventions.
4. **Critically appraise the participant recruitment strategy. (5 marks)**
  - The authors identified potential participants the cohort of patients enrolled in the electronic version, namely the ePEEP telehealth intervention. All patients who had completed the first week of the programme were eligible as week 1 of ePEEP is foundational.
  - When evaluating the recruitment strategy for this study, one must consider both the recruitment for the ePEEP telehealth intervention as well as the subsequent recruitment for the qualitative, exploratory descriptive study, as they are presented together in this article. However, to appraise the recruitment of the participants for the qualitative study component, one has to understand how the authors recruited participants from the cohort of participants who were enrolled in the ePEEP intervention.
  - For the ePEEP intervention, the authors described in the methods section of the article how they approached patients living with chronic pain on the waiting list for the conventional PEEP, to join the telehealth intervention.
  - This recruitment for intervention and the study were performed by separate team members. The recruitment for the ePEEP was carried out by one team member, MZ. This team member also facilitated the contact with the team member who coordinated the study, by contacting patients who completed the ePEEP to ask their permission to share their contact details with the team member, DE, who coordinated the invitations for participation in the research study. Those patients who agreed to being included, received a text message and voice note from DE, which was followed by formal recruitment and informed consent to join the research study.
  - The research team followed a clear process of separate recruitment for the intervention and the linked evaluative study, and both activities were clearly delineated in terms of process and purpose as well as team members who led each activity. This helped to prevent bias and possible role confusion from the perspectives of both the team and patients.
5. **Were the participants included in the study well described? Justify your response. (4 marks)**
  - The authors provide a clear textual description of why only six participants were included in the study in the study participants’ description in the results section. Despite a waiting list for the PEEP intervention of 51 patients, only 27 were meeting the inclusion criteria for the ePEEP groups, and only eleven successfully completed the full programme and who were eligible for the qualitative study.
  - Figure 1 (Ernstzen et al. 2022) provides a complementary visualisation of how this came to be, given the relatively small group of eleven participants who completed all the ePEEP activities, of which one declined and four were not contactable, resulting in the final group of six participants.
  - Table 3 (Ernstzen et al. 2022) provides the age and reported health conditions of the six participants. This table also helps the reader to understand which of the three intervention groups were represented in the study, with only one of the seven participants in group 1 (English speaking), three of the seven participants in group 2 (Afrikaans speaking) and one of the four participants in group 3 (bilingual).
  - In summary, the included participants are reasonably well described by the information presented. The information is not sufficient, however, to assist the reader to contextualise the findings: it is not clear what the ages and reported conditions of the other group members were from which these participants stem. Furthermore, it would have been useful to include additional socio-demographic information of the participants, including household income and educational levels (this information may have been obtained during the interviews but were not reported).
6. **How did the researchers ensure trustworthiness with regard to their approach to qualitative data analysis? Use the following concepts to structure your answer: credibility, transferability, dependability and reflexivity. (8 marks)**

**TABLE 1:** Scoring rubric.

Competencies	Candidate’s rating		
	Not competent	Competent	Good
Establishes and maintains a good doctor-patient relationship	-	-	-
Gathering information: history/examination/investigations	-	-	-
Clinical reasoning	-	-	-
Explanation and planning	-	-	-
Management	-	-	-

The criteria for reporting trustworthiness related to the qualitative data analysis have been described by Lincoln and Guba in 1985 and consist of the following elements, which we may use to evaluate the article:

- Credibility

Several techniques may assist to enhance the credibility of the results, namely prolonged engagement, persistent observation, data collection triangulation and researcher triangulation.

In this article, the authors described the use of member checking (see methods section), by providing the themes and categories to study participants to verify via text message, which was possible given the existing engagement via smartphone in delivering the ePEEP intervention. For this study design, prolonged engagement and persistent observation was not possible. The engagement of participants with the ePEEP intervention group was a theme, but this engagement was not shared by the researchers, as they were independent of the intervention process.

- Transferability

Transferability refers to the generalisability of findings to other contexts, such as that of the reader. This is facilitated by providing thick descriptions of the study setting and participants.

The authors provided a thick description of the PEEP programme offered at the two tertiary hospitals in the introduction section, as well as a sound overview of the ePEEP intervention. The description of the participants in the ePEEP intervention, as well as the sub-group who participated in the qualitative interviews was limited, with only the sub-group’s age and chronic pain condition described. We knew from the ePEEP inclusion criteria that the ePEEP participants had access to a smartphone and had an established diagnosis. Further socio-economic information such household income levels and educational backgrounds were not provided, which limits the transferability of the study findings.

- Dependability

To achieve dependability, researchers can ensure the research process is logical, traceable and clearly documented. An audit trail of the qualitative analysis, including the coding process, helps the reader to understand the researchers’ decisions and choices regarding theoretical and methodological considerations.

The authors provided a clear description of the process followed to divide the six transcripts between three researchers who were not part of delivering the ePEEP intervention. Consensus was reached to form an initial codebook and themes, after which the codebook was applied to the dataset using a qualitative analysis software programme, ATLAS.ti, to help ensure consistent coding of the transcripts. The researchers confirmed that they audited the data analysis procedure, including interview analysis, categories and themes and collaboratively identified associations and relationships between themes. This description of the audit process helps to demonstrate dependability and helps to validate the intercoder reliability.

- Reflexivity

Reflexivity is central to the audit trail. It is important for the reader to understand the research team’s professional background, experience in qualitative research and their relationship with the research question, as well as the study participants and setting.

In this article, the initials of the authors were used to denote the roles of the authors in the recruitment, interview and data analysis steps. The author contributions section also state the contributions to the study and writing the manuscript. The article does not contain a reflexivity report or statement. It is important to observe that the facilitator of the ePEEP intervention was not involved in the qualitative interviews and data analysis process but was also included as a co-author. The professional relationships of the remaining authors with the tertiary hospital pain clinic services have not been well described to the reader. It is important to state the experience and training of the team members, as it helps the reader to gauge the credibility of the researchers, as well as their reasons for doing the research.

### Further reading

- Mabuza LH, Govender I, Ogunbanjo GA, Mash B. African primary care research: Qualitative data analysis and writing results. Afr J Prim Health Care Fam Med. 2014;6(1):1–5. https://doi.org/10.4102/phcfm.v6i1.640
- Nowell LS, Norris JM, White DE, Moules NJ. Thematic analysis: Striving to meet the trustworthiness criteria. Int J Qual Methods. 2017;16(1):1–13. https://doi.org/10.1177/1609406917733847
- Reid S, Mash B. African primary care research: Qualitative interviewing in primary care. Afr J Prim Health Care Fam Med. 2014;6(1):1–6. https://doi.org/10.4102/phcfm.v6i1.632
- The Joanna Briggs Institute. Critical appraisal tools [homepage on the Internet]. The Joanna Briggs Institute; 2023 [cited 2023 Oct 31]. Available from: https://jbi.global/critical-appraisal-tools
- Tong A, Sainsbury P, Craig J. Consolidated criteria for reporting qualitative research (COREQ): A 32-item checklist for interviews and focus groups. Int J Qual Health Care. 2007;19(6):349–357. https://doi.org/10.1093/intqhc/mzm042
- Williams V, Boylan AM, Nunan D. Critical appraisal of qualitative research: Necessity, partialities and the issue of bias. BMJ Evid Based Med. 2020;25(1):9–11. https://doi.org/10.1136/bmjebm-2018-111132

## Objectively structured clinical examination (OSCE) station: Anaesthesia

### Objective of station

This station tests the candidate’s ability to adapt anaesthesia techniques and communication for a paediatric patient, considering the unique physical and emotional needs of children and their families. It also evaluates their capacity to provide safe and effective care in a paediatric setting, which is crucial in paediatric anaesthesia.

### Requirements

- Simulated patient: 6-year-old child, parents of the child

### Instructions for candidate

You are the family physician working at a district hospital and you have been assigned to cover Anaesthesiology this morning. You are called to the pre-operative area to assess and prepare a 6-year-old patient scheduled for a tonsillectomy and adenoidectomy. The child is anxious and so are the parents who are concerned about the child’s well-being during anaesthesia and surgery

Your task is to adapt anaesthesia techniques and communication effectively for this paediatric patient. You have 15 min to complete this station.

### Instructions for the examiner

This is an integrated consultation station in which the candidate has 15 min. Familiarise yourself with the assessor guidelines (Table 1), which details the required responses expected from the candidate.

No marks are allocated. In the mark sheet, tick off one of the three responses for each of the competencies listed. Make sure you are clear on what the criteria are for judging a candidates’ competence in each area.

### Further reading

- Straus L. Anaesthetic management of paediatric adenotonsillectomy. S. Afr Fam Pract. 2012;54(3):S17–S20. https://doi.org/10.1080/20786204.2012.10874231
- Bangera A. Anaesthesia for adenotonsillectomy: An update. Indian J Anaesth. 2017;61(2):103–109. https://doi.org/10.4103/0019-5049.199855

### Guidance for examiners

The aim is to establish that the candidate has an effective and safe approach to adapting anaesthesia techniques to a paediatric patient and communicating effectively to allay fears and anxiety in the child patient and the parents.

Anaesthesia for adenotonsillectomy is a challenge to the anaesthesiologist. A safe conduct of anaesthesia is of utmost importance to avoid the complications and associated anxiety of the patient as well as the parents. Good communication between the surgeon, anaesthesiologist and the parents of the child is a must for a successful outcome.

The goals are adequate patient preparation with premedication, providing good surgical access while ‘sharing the airway’, optimising perioperative analgesia, preventing post‑operative nausea and vomiting, perioperative airway management and prevention and timely management of post‑operative haemorrhage and other complications.

*Not competent:* Patient safety is compromised (including ethico-legal considerations) or task is not completed

*Competent*: The task is completed safely and effectively

*Good*: In addition to displaying competence, the task is completed efficiently and in an empathic, patient-centred manner (acknowledges patient’s ideas, beliefs, expectations, concerns/fears)

1. Establishes and maintains a good doctor–patient relationship

The competent candidate is respectful, engages with the patient in a dignified and caring manner.

(*Approach the child and parents in a child friendly manner to establish rapport and alleviate anxiety. Begin with a warm and friendly introduction maintaining eye contact with the patient and her parents, Address patient by her name, introduce yourself letting her know your role as the ‘doctor who helps children fall asleep for surgery’*)

The good candidate is empathic, compassionate and collaborative, facilitating active participation in key areas of the consultation.

(*Can say ‘It’s okay to feel a little scared, but we are here to make everything safe and comfortable for you’, Offer reassurance by telling the child that her parents will be with her before and after the procedure; Maintains the calm, friendly and empathic approach throughout the consultation*)

2. Gathering information: history/examination/investigations

The competent candidate gathers sufficient information to establish a clinical assessment (*Elicit a relevant and detailed history including underlying medical conditions or chronic illnesses, previous surgeries or hospitalisations, medications, known allergies; any history of difficult airway management or complications during previous anaesthesia if relevant; developmental stage and any developmental delays; while conducting a thorough assessment of the paediatric patient including vital signs, airway assessment and physical examination*).

The good candidate additionally has a structured and holistic open approach with child and the parents (*Explore circumstances that led to the surgical decision with respect to Individual and Contextual issue; Elicit information about child’s temperament and behaviour concerns and emotional and psychological factors that may affect her response to anaesthesia*).

Top of Form

3. Clinical reasoning

The competent candidate selects the appropriate anaesthetic technique based on the patient’s needs and the surgical procedure

(*Decide on inhalational anaesthesia or intravenous anaesthesia or combination, recognise the anxiety in the patients and her parents*)

The good candidate has a structured approach to address the patient’s agenda (*Justify the choice of technique; develops a comprehensive anaesthetic plan tailored to the patient’s age, size and procedure*)

4. Explanation and planning

The competent candidate uses clear language and information to explain to the patient and her parents and uses strategies to ensure patient understanding.

(*Explain reason for the surgery and why anaesthesia is necessary Can say: you are here to have your tonsils and adenoids removed because they might be causing problems with your breathing, sleeping and/or the reason for your frequent episodes of sore throat communicate the anaesthesia process and what to expect to the patient and the parents, ensuring an age appropriate language*)

*Explain that anaesthesia can be given in different ways, such as through a special mask (if using inhalation anaesthesia) or through an IV (if using intravenous anaesthesia). Use familiar and non-threatening terms like ‘magic mask’ or ‘sleepy juice’ to describe the equipment or medications; addressing the concerns of the parents by allowing them to ask questions*

The good candidate additionally ensures that the child and parents are actively involved in decision-making, paying particular attention to knowledge-sharing and empowerment,

(*Invite the child to ask any questions they might have. Reassure them that there are no ‘silly’ questions, and you’re there to answer and address their concerns. Listen attentively and provide honest and age-appropriate answers. Reassure the child and their parents that their safety is the top priority. Explain that anaesthesia will help them to sleep comfortably and painlessly during the surgery.*)

5. Management

Proposes appropriate intervention with anaesthesia induction, plan airway management, intraoperative care and monitoring, emergency and post-operative care, pain management and discharge planning. Discuss with the parents:

*A secure and age-appropriate airway management strategy will be employed to ensure safe and effective ventilation.*

*Throughout the surgery, child will be continuously monitored to ensure her safety and well-being.*

*Her vital signs, including heart rate, blood pressure, oxygen saturation and end-tidal CO*_*2*_, *will be closely tracked.*

*Anaesthesia depth and medications will be adjusted to maintain appropriate anaesthesia levels.*

*After the surgical procedure is complete, the child will gradually wake up from anaesthesia in the post-anaesthesia care unit*

*Her vital signs, including respiratory function and oxygen saturation, will be closely monitored.*

*Adequate pain management strategies will be employed to ensure Emma’s comfort during the recovery period. This may include pain medications or regional anaesthesia techniques.*

The good candidate additionally discusses a discharge plan

(*Child’s parents will be allowed to be with her as she wakes up, providing comfort and reassurance.*

*Child will only be discharged when she has fully recovered from anaesthesia, is stable and meets predefined criteria for discharge.*

*Detailed postoperative care instructions will be provided to her parents or caregivers.*)

**Role play – Instructions for actor**

- Child 6 years old – has not eaten since midnight as per preoperative fasting guidelines
- Mother 28 years – legal assistant at Law firm
- Father 32 years – Teacher

Opening statement: ‘Mummy, I am scared … I don’t want to stay here, I want to go home …

Mother – Doctor please tell us what is going to happen, we were just told that my baby has to have her tonsils removed. Will she feel any pain? What if she wakes up during the operation; Will she be fine?

Father – Doc I’m leaving my child in your hands, I don’t want any mess ups …

Child – 6 years, RVD unexposed history of repeated episodes of tonsillitis associated with pyrexia, almost 8 episodes in the last year. Last episode 1 week ago …

Uneventful birth, 3.2 kg currently 18 kg, immunisation is up to date

Siblings: none

Father smoker, no chronic diseases

Heard about problems in the hospitals regarding anaesthetics. Really worried about negligence

Mother, Type 1 diabetic on insulin, extremely distressed, this was a precious baby conceived after much difficulty because of her diabetes

No significant family history

Examination findings

- Body mass index: 19 kg/m^2^
- Blood pressure: 118/72 mmHg, heart rate: 112 beats/min
- Haemoglobin: 14.5 gm/dL
- Random blood glucose (HGT): 5.9 mmol/L
- Urinalysis: No abnormalities
- Ear, nose and throat: Normal
- Cardio-respiratory systems: No abnormalities
- Abdomen: No abnormalities
- Neuro: No abnormalities

